# Kinetics and Disappearance of QRS Transition in Patients Undergoing Left Bundle Branch Pacing – A Novel Method for Classifying Microdislodgement

**DOI:** 10.1111/jce.16779

**Published:** 2025-07-11

**Authors:** Márk Gémesi, Balázs Polgár, Zoltán Gingl, István Marczell, Előd János Zsigmond, Letícia Chityil–Papp, Péter Bógyi, Gábor Zoltán Duray

**Affiliations:** ^1^ Department of Cardiology Central Hospital of Northern Pest ‐ Military Hospital Budapest Hungary; ^2^ Faculty of Medicine Semmelweis University Budapest Hungary; ^3^ Doctoral College Semmelweis University Budapest Hungary; ^4^ Heart and Vascular Centre Semmelweis University Budapest Hungary

**Keywords:** left bundle branch area pacing (LBBAP), left bundle branch pacing (LBBP), left ventricular septal pacing (LVSP), microdislodgement, QRS transition

## Abstract

**Introduction:**

QRS transition during the threshold test is the gold standard for confirming direct capture of the Conduction System in patients with left bundle branch pacing (LBBP). Still, we have limited data on the kinetics of QRS transition over time. Microdislodgement is a known complication of left bundle branch area pacing (LBBAP); however, its true incidence depends on the thoroughness of follow‐up. We aimed to evaluate the kinetics and disappearance of QRS transition in patients undergoing LBBP, assess the diagnostic yield of QRS transition at various time points, and characterize microdislodgement.

**Methods:**

This prospective study included patients who underwent successful LBBP procedures at a tertiary center between January 2022 and February 2024. Based on the kinetics of QRS transition during intraoperative, postoperative, and follow‐up threshold tests, microdislodgement was assessed.

**Results:**

LBB capture was confirmed in 118 of 155 LBBAP patients (76.1%), which defined our LBBP population. Intraoperative QRS transition was observed in 86.4%, which decreased significantly postoperatively (47.0%) and at follow‐up (33.0%)—in 92.0% of LBBP patients' capture of LBB remained, while microdislodgement occurred only in 5.0% of LBBP cases.

**Conclusion:**

This study evaluates QRS transition kinetics in LBBAP patients, showing significant intraoperative detectability in patients with direct capture of LBB that decreases postoperatively and at first follow‐up. Our classification of microdislodgement aids in understanding its impact on pacing outcomes.

## Graphical abstract

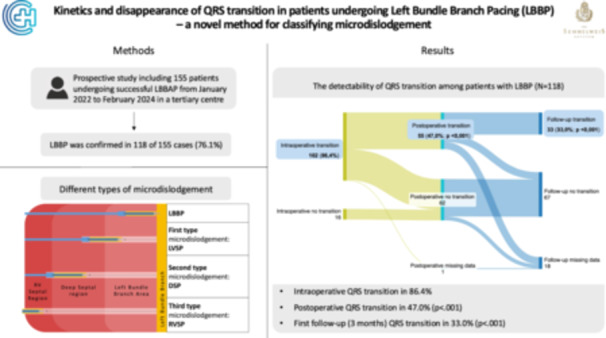

## 1 Introduction

Left bundle branch area pacing (LBBAP) is a safe and feasible pacing method [1, 2, 3, 4, 5, 6, 7]. Its clinical advantages over his bundle pacing (HBP) rely on the larger anatomical target area and the more favorable electrical parameters [4, 8, 9, 10]. However, long‐term lead performance data regarding LBBAP are still lacking. From the perspective of the Left Conduction System capture, two main subgroups can be distinguished: (a) through direct activation of left bundle branch (LBB), left bundle trunk (LBTP), or left fascicular pacing (LFP), overall left bundle branch pacing (LBBP) can be achieved, (b) through the capture of the left subendocardial myocardium left ventricular septal pacing (LVSP) can be performed, without direct activation of the Conduction System [11, 12, 13]. Compared to HBP, the differentiation between the capture types of LBBAP is more difficult, and its clinical significance is still not clarified. In terms of LBBP, LFP is much more common than LBTP and is more achievable and as beneficial as LBTP in terms of electrical synchrony and pacing reliability [12, 14].

According to the 2023 EHRA Consensus Statement Document, the gold standard method to confirm the direct capture of the Conduction System is the presence of transition in QRS morphology during the threshold test, which has a higher diagnostic yield when performed immediately after lead fixation [13]. Based on a current study, the presence of QRS transition independently predicts a greater improvement in left ventricular ejection fraction (LVEF) among patients with LBBAP resynchronization therapy [15].

To date, studies investigating the detectability of QRS transition have reported inconsistent results. Su et al. reported that QRS transition was detectable in 75.4% of cases demonstrating s‐LBBP, and only 30.9% of patients showed selective transition at 3 months follow‐up [16]. However, based on a recent study by Shimeno et al., QRS transition remained consistently reproducible during follow‐up. During a 1‐year follow‐up, QRS transition was observed in 88% of LBBAP patients who experienced intraoperative QRS transition [17].

Lead microdislodgement is a known complication of the conduction system pacing (CSP). It is defined by the loss of capture type achieved at implantation. Microdislodgement can occur periprocedurally or during follow‐up and is considered an underreported event [13]. Loss of LBBAP, which is characterized by the disappearance of the terminal r/R' wave in V1, was observed in 4.0% of the MELOS study population [12]. The different types of microdislodgement have not been classified yet.

The aim of our study was (a) to evaluate the presence of QRS transition intraoperatively, postoperatively, and at the first follow‐up among patients receiving LBBP; (b) to investigate possible reasons behind the disappearance of the loss of QRS transition at any point during follow‐up; (c) to characterize in specific subtypes of microdislodgement.

## 2 Methods

The study was conducted at the Central Hospital of Northern Pest ‐ Military Hospital (Budapest, Hungary), at a tertiary center, including patients who underwent LBBAP implantation, after its approval had been granted by the Institutional Ethics Committee. Patients were prospectively enrolled between January 2022 and February 2024.

### 2.1 Patient Selection

The study population comprises all consecutive patients who underwent LBBAP at this center. Indication of implantation consisted mainly of bradycardia with expected high ventricular pacing burden and/or decreased LVEF, “pace‐and‐ablate,” and resynchronization indications. Upgrade indications were also included. Depending on the indication or anatomy, we aimed for LBBP or settled for LVSP.

### 2.2 Implantation Procedure

Procedures were performed by using Medtronic SelectSecure 3830 lumenless active fixed helix leads or Biotronik Solia S60 style‐driven leads (SDL). The LBBAP implantation procedure was routinely performed using the previously described methods by three physicians of this hospital [2, 12, 13, 18, 19, 20].

Firstly, the tricuspid annulus was localized with contrast injection. Secondly, the sheath was positioned distally from the tricuspid valve, and pacemapping was performed at the right side of the interventricular septum. A favorable paced QRS for the penetration site can be described with QS and notched nadir in V1, resembling a “W pattern.” Discordant QRS polarity in lead II and III was accepted for fixation, as well as QS complexes or R waves in lead II and III. At this site, the lead penetrated the RV septum by rapid rotations. Recent data have shown that tricuspid regurgitation is more common when the pacing lead is positioned proximal to the tricuspid valve summit [21]. Therefore, we aimed to achieve a more distal LBBAP, differing from the clinical practice mentioned above. During the screw‐in process, fluoroscopy, paced QRS morphology, presence of fixation beats, pacing threshold, and lead impedance were monitored to determine the adequate depth of the pacing lead.

### 2.3 Threshold Test

The threshold test is described as a decremental voltage output pacing performed in unipolar mode to avoid anodal capture. By gradually decreasing the stimulating output voltage, depending on the electrode position, we can observe a transition in QRS morphology to either selective capture of the LBB (s‐LBBP) or LVSP. All threshold tests were performed in unipolar pacing mode at all three time points: at the end of the implantation, at the 16–72‐h postoperative observation period, and at the first follow‐up visit, which was performed 3 months after the procedure.

### 2.4 Definition of LBBAP, LBB Capture Criteria

LBBAP is a practical terminology when the pacing lead penetrates the left subendocardial region, with or without the direct capture of the LBB [13]. This definition rests on the morphology of the paced QRS; terminal R‐wave pattern can be observed in lead V1 during stimulation of the left bundle branch area (LBBA).

In our study, LBB capture was verified if we managed to enter the LBBA, in addition to one of the following criteria [13, 20, 22, 23, 24, 25]:

a. The diagnostic transition from ns‐LBBP to s‐LBBP during the threshold test could be detected,
  - the appearance of M/rsR' pattern and wide R' with a notch in lead V1
  - S wave in V5/V6 with constant V6RWPT
b. The diagnostic transition from ns‐LBBP to LVSP during the threshold test could be detected,
  - abrupt prolongation V6RWPT at least 10 ms
c. Measurement of the V6RWPT,
  - in patients with narrow QRS/isolated RBBB, V6RWPT < 75 ms
  - in patients with LBBB, NIVCD, and wide QRS escape rhythm, V6RWPT < 80 ms
d. V6‐V1 interpeak interval > 44 ms,
e. Potential‐V6RWPT = stimulus‐V6RWPT, allowing a variability with less than 10 ms.

If none of the LBBP criteria were detected during the assessment, and the LBBA was previously confirmed, then LVSP was verified. At follow‐up, if QRS transition was no longer detectable, LBB capture was considered preserved if the paced QRS morphology, V6RWPT, and V6‐V1 interpeak interval remained unchanged compared to intraoperative values.

In patients with bradycardia and preserved LVEF, Conduction System capture was not systematically targeted by the implanting physicians. This decision was based on the current lack of evidence supporting a clear clinical benefit of CSP in this subgroup, considering that attempting to achieve Conduction System capture may increase the risk of septal transfixation [26]. Therefore, in such cases, LVSP was accepted as the final pacing strategy when LBB capture criteria were not met.

### 2.5 Determination of the LBB Pacing Site

Previously described criteria were used to determine the pacing site of the LBB [12, 27, 28]. The presence of LBB or fascicular potentials, together with the QRS axis in the frontal plane, was analyzed and the potential‐ventricular electrogram (vEGM) interval was measured. The different types of stimulation sites were evaluated as below.

- LBTP: QRS axis similar to the intrinsic sinus rhythm and potential‐vEGM interval of 25–35 ms.
- Left anterior fascicular pacing (LAFP): dominant S wave in leads I and aVL, dominant R wave in inferior leads, with fascicular potential‐vEGM interval < 25 ms or without potential.
- Left septal fascicular pacing (LSFP): QRS axis like sinus rhythm or inferior axis with a negative component in lead III, with fascicular potential‐vEGM interval < 25 ms or without potential.
- Left posterior fascicular pacing (LPFP): dominant R wave in leads I and aVL, dominant S wave in inferior leads, with fascicular potential‐vEGM interval < 25 ms or without potential.

### 2.6 Microdislodgement

**Figure 1:**
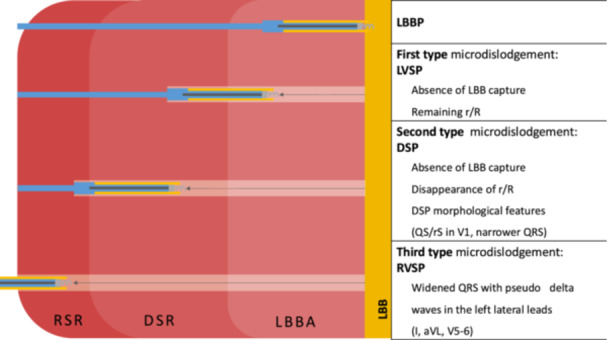
Differentiation of microdislodgement. Electrocardiographic parameters of each microdislodgement type are detailed on the right side of the figure, with the corresponding anatomical lead positions on the left. First‐type microdislodgement can be characterized as LVSP. Second‐type microdislodgement can be identified as DSP. Third‐type microdislodgement can be described as RVSP. DSP, deep septal pacing; DSR, deep septal region; LBB, left bundle branch; LBBA, left bundle branch area; LBBP, left bundle branch pacing; LVSP, left ventricular septal pacing; RSR, right septal region.

We defined three different types of microdislodgements, detailed in Figure 1. Each subtype was classified according to the location of the lead in the septum and consequently the stimulated ECG pattern:

- First type microdislodgement: characterized by the loss of LBB capture, with remaining terminal r/R' in V1, indicating LVSP.
- Second type microdislodgement: Identified by the loss of LBB capture and loss of terminal r/R' in V1, reflecting a pacing site deep within the septum that does not engage the left ventricular subendocardial area (LBBA). Deep septal pacing (DSP) morphological features, such as narrower QRS or QS or rS morphology in V1.
- Third type microdislodgement: Marked by widened QRS complex with pseudo‐delta waves in the left lateral leads (I, aVL, V5‐6). The ECG pattern is consistent with right ventricular septal pacing (RVSP), leading to a broader and less synchronous ventricular contraction.

If QRS transition during the threshold test was lost over time, microdislodgement was excluded by unchanged QRS morphology, V6RWPT, and V6V1 interpeak interval compared to operative data.

### 2.7 Data Collection

The demographic data, medical history, and preoperative electrocardiography of all LBBAP patients were collected at enrollment. In each patient, procedure‐related characteristics such as native‐ and paced twelve‐lead electrocardiograms (ECGs), fluoroscopy exposure time, and duration of the procedure were recorded during the implantation. During the assessment of the LBB capture, the previously mentioned electrocardiographic parameters were measured immediately after lead fixation and controlled after the slitting phase of the procedure. Additionally, decremental output threshold tests were performed during the 16–72 h postimplantation phase and at the first follow‐up visit (3 months after the procedure). If the QRS transition was not detectable at any time in our study period, then the LBB capture was determined using other diagnostic methods mentioned above.

### 2.8 Safety Endpoints

Data on major intraoperative acute procedure‐related adverse events, including pneumothorax, cardiac tamponade, or other arrhythmic events, were collected among LBBAP patients. Additionally, minor intraoperative complications, including septal perforation and micro‐ or macrodislodgement during the implantation, are assessed in the LBBAP cohort. Device and lead‐related complications, such as micro‐ and macrodislodgement or any other electrode malfunction at any time until the 3‐month follow‐up, were recorded among patients receiving LBBP.

### 2.9 Statistical Analysis

Data management and analyses were performed with SPSS, version 29.0 (SPSS). Continuous variables are presented as mean ± standard deviation (SD). For group comparisons, the independent samples Student's *t*‐test was used for normally distributed variables, while the Mann–Whitney *U* test was applied for variables with non‐normal distribution. Categorical variables are expressed as frequencies and percentages, and were compared using the Chi‐square test or Fisher's exact test, as appropriate. A *p*‐value of < 0.05 was considered statistically significant.

## 3 Results

### 3.1 Baseline Characteristics and Procedural Parameters of the LBBAP Population

Between January 2022 and February 2024, a total of 171 patients were scheduled for LBBAP, and in 155 patients (90.6%), implantation was successfully carried out, meeting the criteria for LBBAP. Baseline characteristics and procedural details of the LBBAP population are presented in Table 1. The mean age of the patients was 72.6 ± 10.9 years, and 70.3% were male. The LVEF at baseline was 46.7 ± 14.3%. The predominant pacing indication was atrioventricular (AV) block (60.0%). In 142 of 155 patients (91.6%), de novo pacemaker implantation was performed, while in the remaining 13 cases (8.4%), revision of the previous pacemaker system was carried out. Most procedures utilized stylet‐driven leads (66.5%), with an average procedural time of 63.9 ± 33.9 min, from first incision to last suture. The mean radiation time was 6.9 ± 8.7 min.

**Table 1:** Baseline characteristics of the successfully implanted LBBAP patient population (*N* = 155).

Clinical variable	
Age (years)	72.6 ± 10.9
BMI (kg/m^2^)	29.2 ± 4.8
Male	109 (70.3)
Hypertension	129 (83.2)
Diabetes mellitus	60 (38.7)
AF	75 (48.4)
ICM	57 (36.8)
CKD*	55 (35.5)
COPD	21 (13.5)
Stroke/TIA	17 (11.0)
NYHA class	1.93 ± 1.05
**Pacing indication**	
AV block	93 (60.0)
Sinus node disease	8 (5.2)
Binodal disease	17 (11.0)
Atrial tachyarrhythmia and planned AVN ablation	11 (7.1)
Primary CRT	13 (8.4)
Upgrade	13 (8.4)
**Echocardiographic parameters**	
LVEF (%)	46.7 ± 14.3
LVEF < 40%	51 (33.6)
LVEDD (mm)	52.6 ± 7.2
LVESD (mm)	36.8 ± 9.1
LA (mm)	55.6 ± 7.8
RA (mm)	55.7 ± 7.2
TR (grade)	1.14 ± 0.66
PASP (mmHg)	39.2 ± 15.3
TAPSE (mm)	19.6 ± 5.1
**Baseline ECG characteristics**	
PQ interval (ms)	212.4 ± 62.8
Native QRS width (ms)	121.4 ± 33.6
Wide QRS (> 120 ms)	66 (42.6)
**QRS morphology (*n*** = **153)**	
Isolated RBBB	24 (15.7)
LFB	13 (8.5)
RBBB + LFB	13 (8.5)
LBBB	35 (22.9)
Alternating LBBB and RBBB	2 (1.3)
NIVCD	1 (0.7)
Asystole/PM dependent	10 (6.5)
**Type of LBBAP lead**	
Lumenless	52 (33.5)
Stylet‐driven	103 (66.5)
**Implantation details**	
Duration (min)	63.6 ± 33.9
Fluoroscopy (min)	6.9 ± 8.7
Contrast agent (mL)	30.6 ± 26.0
**Electrical parameters (acute setting)**	
R wave sensing (mV)	10.2 ± 6.1
Impedance (Ω)	726 ± 174
Threshold (V) (×0.4 ms)	1.04 ± 0.47
**Type of implanted device**	
VVI pacemaker	22 (14.2)
VVI‐ICD	1 (0.6)
DDD pacemaker	106 (68.4)
DDD‐ICD	2 (1.3)
CRT‐P	14 (9.1)
CRT‐D	10 (6.5)

*Note:* Values are given as mean ± SD or *n* (%).

Abbreviations: AF, atrial fibrillation; AV, atrioventricular; BMI, body mass index; CKD, chronic kidney failure; COPD, chronic obstructive pulmonary disease; CRT, cardiac resynchronization therapy; CRT‐D, cardiac resynchronization therapy – implantable cardioverter‐defibrillator; CRT‐P, cardiac resynchronization therapy – pacemaker; ECG, electrocardiogram; ICD, implantable cardioverter defibrillator; ICM, ischemic cardiomyopathy; LA, left atrium; LBBB, left bundle branch block; LBBAP, left bundle branch area pacing; LFB, left fascicular block; LVEDD, left ventricular end‐diastolic diameter; LVESD, left ventricular end‐systolic diameter; LVEF, left ventricular ejection fraction; NIVCD, nonspecific interventricular conduction disease; NYHA HF, New York Heart Association classification for heart failure; PASP, pulmonary arterial systolic pressure; PM, pacemaker; RA, right atrium; RBBB, right bundle branch block; TAPSE, tricuspid annular plane systolic excursion; TR, tricuspidal regurgitation; TIA, transient ischemic attack.

* Glomerular filtration rate < 60 mL/min/1.73 m^2^.

### 3.2 Electrophysiological Characteristics of the LBBAP Population

According to the previously detailed criteria, direct capture of LBB was confirmed in 118 of 155 patients (76.1%). In contrast, LVSP was verified in the remaining 37 cases (23.9%) based on the absence of LBB capture criteria. The previously described stepwise algorithm was used as diagnostic criteria for LBB capture (Supporting Information S1: Figure S1. Procedural electrophysiological characteristics for both pacing types, such as final QRS duration, V6RWPT, and V6‐V1 interpeak interval, are detailed in Table 2. In terms of LBBP subtypes, LSFP was the most common type of fascicular capture (43.2%), followed by LPFP (41.1%) and LAFP (15.3%).

**Table 2:** Electrophysiological characteristics of the study population (*N* = 155).

	LBBP (*n* = 118)	LVSP (*n* = 37)	*p* value
Final QRS duration (*n* = 152)	127.7 ± 22.6	136.9* ± *27.1	0.046
V6RWPT (*n* = 155)	72.9 ± 13.1	85.6* ± *15.9	**< 0.001**
V6‐V1 interpeak interval (*n* = 147)	43.2 ± 16.2	40.8* ± *17.0	0.458

*Note:* Values are given as mean ± SD.

Abbreviations: LBBP, left bundle branch pacing; LVSP, left ventricular septal pacing; V6RWPT, V6 R wave peak time.

### 3.3 Intraoperative Complications of the LBBAP Population

Intraoperatively, ventricular tachycardia (VT) was detected in one case (0.6%). We observed septal perforation in nine cases (5.8%) and atrial macrodislodgement in one case (0.6%). In terms of the Conduction System lead, any type of microdislodgement in six cases (3.9%) and macrodislodgement in one case (0.6%) occurred at the implantation. In these cases, lead repositioning was performed intraoperatively to reach LBBAP. Each microdislodgement arose during the slitting of the sheath.

### 3.4 QRS Transition Among Patients Receiving LBBP

**Figure 2:**
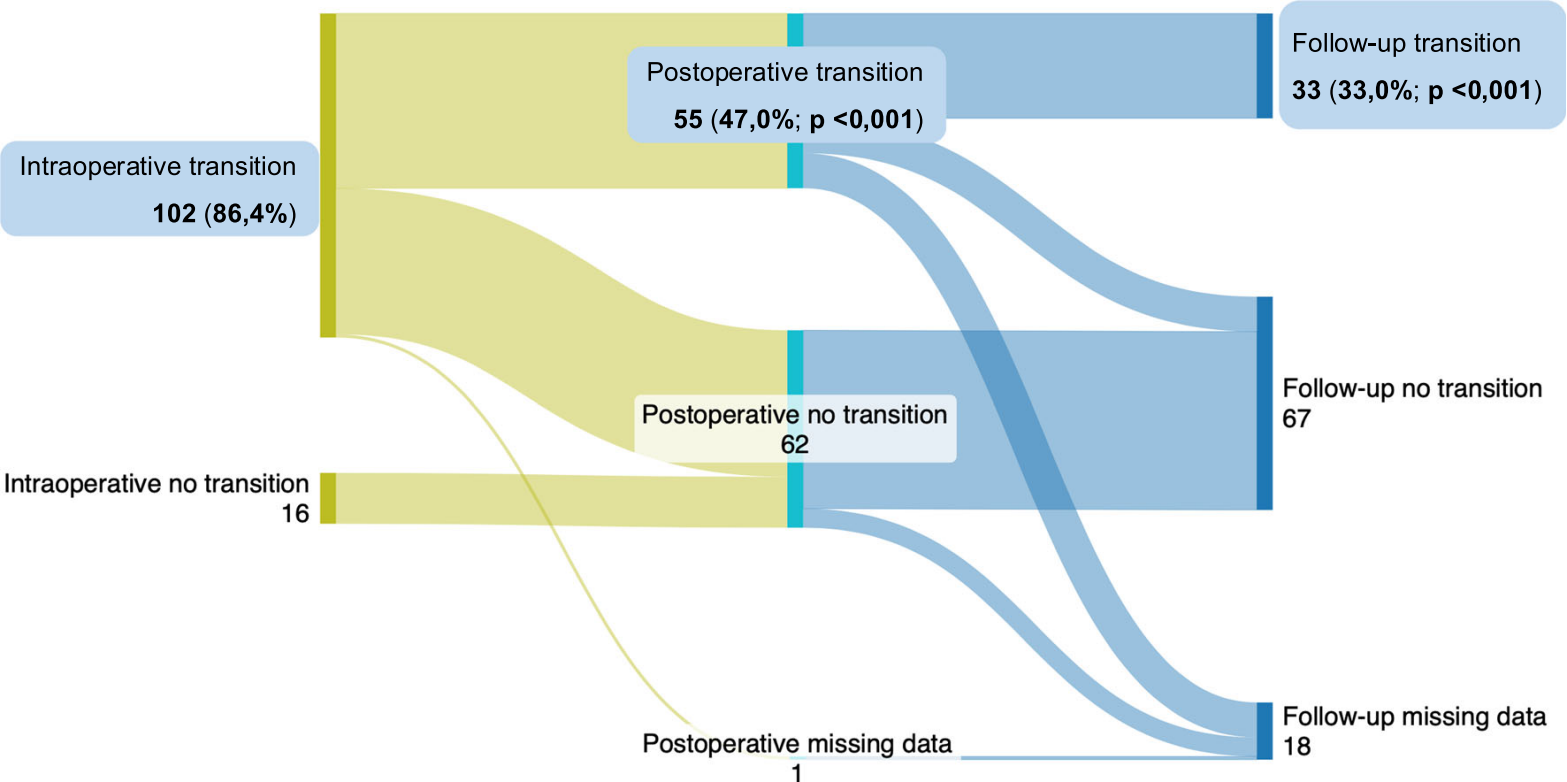
The detectability of QRS transition intra‐, postoperatively, and during follow‐up (*N* = 118.) The detectability of QRS transition is presented intraoperatively, postoperatively, and during follow‐up in the LBBP population (*N* = 118). “Transition” means the amount of detectable‐, “no transition” means the amount of not detectable QRS transition during the decremental threshold test. Values are given as *N*.

In 102 of 118 patients (86.4%), QRS transition from ns‐LBBP to either s‐LBBP or LVSP was detected intraoperatively; in 75 of 118 LBBP patients (67.6%), QRS transition from ns‐LBBP to s‐LBBP‐; and in 20 of 118 LBBP patients (18.0%), QRS transition from ns‐LBBP to LVSP was detected during the intraoperative threshold test. In contrast, transition was not observed in 16 of 118 LBBP cases (14.4%) intraoperatively. We managed to perform threshold tests in 117 of 118 cases (99.2%) postoperatively, and in 100 of 118 cases (84.7%) during the first follow‐up (Figure 2).

**Figure 3:**
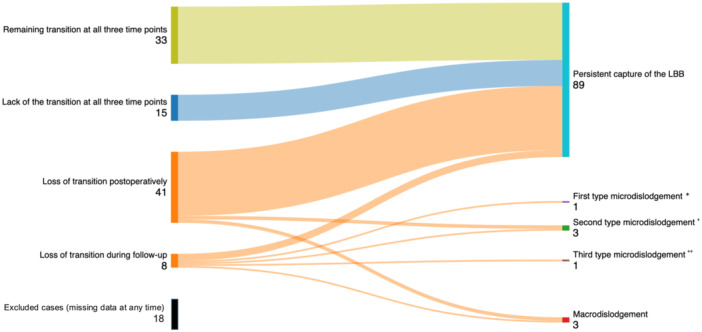
Loss of QRS transition postoperatively and during follow‐up among patients with LBBP with three‐time point threshold test (*N* = 118). In this figure, the disappearance of QRS transition is presented postoperatively and during follow‐up among patients with LBBP with a three‐time point threshold test supplemented with detected possibilities. Cases in which the threshold test was not performed at any time were excluded from the analysis of causes of QRS transient disappearance to avoid bias. Values are given as *N*. *No presence of LBB capture with terminal r/R' in V1. ^+^No presence of LBB capture without terminal r/R' in V1 lead, DSP morphological features can be detected. ^++^Wide QRS with pseudo‐delta waves in the left lateral leads (I, aVL, V5‐6), RVSP.

The detectability of the QRS transition is shown in Figure 3. During the repeated threshold test between the 16–72 h postimplantation phase, the transition was detected in only 55 of 117 cases (47.0%; *p* < 0.001), which is a significant reduction in contrast to the intraoperative data. Three months after the procedure, at the first follow‐up, QRS transition was confirmed in only 33 of 100 patients (33.0%; *p* < 0.001), which was significantly less frequent compared to the postoperative measurements.

To evaluate the potential impact of cases with missing data at any time points, a sensitivity analysis was performed comparing baseline and procedural characteristics between patients with complete (all three time point threshold test data) and incomplete data (missing data at any time point; Supplementary Table S1). No statistically significant differences were found in age, sex, LVEF, QRS width, comorbidities, or implantation duration, indicating that excluding these cases is unlikely to have introduced systematic bias.

We did not find statistical significance between the type of pacing leads and the detectability of QRS transition, neither intraoperatively (*p* = 0.774), nor postoperatively (*p* = 0.240), nor during follow‐up (*p* = 0.655).

### 3.5 Lead‐Related Complications During Follow‐Up Among Patients With LBBP

In Table 3, we have listed all lead‐related complications observed during the postoperative monitoring period of 16–72 h or up to the 3‐month follow‐up. Among these complications, the following resulted in the disappearance of the QRS transition: any type of microdislodgement in 5 of 118 cases (4.2%), macrodislodgement in 3 of 118 cases (3.4%), either at the postoperative observation or during follow‐up.

**Table 3:** Perioperative complications of study population (*N* = 155).

		Intraoperative	Postoperative
Septal perforation		9 (5.8)	0 (0.0)
Microdislodgement	First type (LVSP)^a^	2 (1.3)	0 (0.0)
	Second type (DSP)^b^	3 (1.9)	2 (1.3)
	Third type (RVSP)^c^	1 (0.6)	0 (0.0)
	All types of microdislodgement	6 (3.9)	2 (1.3)
Macrodislodgement		1 (0.6)	2 (1.3)
VT		1 (0.6)	1 (0.6)
RA macrodislodgement		1 (0.6)	1 (0.6)
Pneumothorax		0 (0.0)	1 (0.6)
Cardiac tamponade		0 (0.0)	1 (0.6)

*Note:* Values are given as *N* (%), numbers are not mutually exclusive.

Abbreviations: DSP, deep septal pacing; RA, right atrium; RVSP, right ventricular septal pacing; VT, Ventricular tachycardia.

^a^ No presence of LBB capture with terminal r/R' in V1 lead.

^b^ No presence of LBB capture without terminal r/R' in V1 lead, DSP morphological features can be detected.

^c^ Wide QRS with pseudo‐delta waves in the left lateral leads (I, aVL, V5‐6), RVSP.

### 3.6 Causes of Transition Disappearance Postoperatively and During Follow‐Up Among Patients With LBBP

We investigated the causes of QRS transition disappearance in these patients, as detailed in Figure 3. Cases in which the threshold test was not performed at any time were excluded from the analysis of causes of QRS transient disappearance to avoid bias. In total, this was found in 18 of 118 LBBP cases (15.3%). The presence of QRS transition disappeared in 52 of 100 LBBP cases (52.0%) over time. Disappearance of QRS transition occurred in 41 of 100 cases (41.0%) postoperatively and in 11 of 100 cases (11.0%) during the first follow‐up. Despite the loss of the QRS transition (52 of 100 cases; 52.0%), the capture of the LBB was persistent over time in 92 of 100 cases (92.0%), while in a minority of cases, the disappearance of the QRS transition was caused by lead‐related complications; microdislodgement in 5 of 100 cases (5.0%) and macrodislodgement in 3 of 100 cases (3.0%).

## 4 Discussion

The primary objective of this study was to evaluate the presence of transition in patients undergoing LBBP and to characterize microdislodgement accurately. Our findings provide important insights into QRS transition as an indicator of the effective capture of LBB.

Several studies have demonstrated the superiority of LBBP over LVSP and BIVP in CRT indication, highlighting its benefits in synchrony, hemodynamics, and clinical outcomes. Diaz et al. found that LBBP led to significantly better LV synchrony and hemodynamic status than LVSP and BIVP, with a superior primary composite outcome, including lower heart failure hospitalization and mortality rates [29]. Curila et al. showed that LBBP and LVSP achieved at least comparable, if not superior, LV synchrony and contractility compared to BIVP. Their findings emphasize that LBBP allows for more natural ventricular activation, potentially explaining its superior hemodynamic benefits in CRT [30]. Zhu et al. demonstrated that LBBP was associated with better long‐term outcomes, including lower mortality and reduced heart failure hospitalizations compared to LVSP and BIVP [31]. Overall, the advantage of directly capturing LBB may be substantial in resynchronization therapy, as LBBP is linked not only to improved LV synchrony and hemodynamic status but also to a better clinical outcome compared to LVSP and BIVP [28, 29, 30].

### 4.1 Advantages and Limitations of the Methods Confirming Direct Capture of LBB

There are several methods to confirm the direct capture of LBB; however, most of them have limitations. Therefore, a complex evaluation of these parameters and tests is important not only during the procedural phase but also during follow‐up.

QRS transition during the threshold test is the gold standard method to evaluate direct capture of the LBB. The biggest advantage over other methods is that the presence of the transition is diagnostic itself.

As a result of lead insertion, a transient increase in excitability potential occurs in the myocardium due to current of injury (COI). The cause of QRS transition lies in the different excitability of this myocardium and the Conduction System, which is influenced by the acute myocardial COI. Consequently, transition during the threshold test has a higher diagnostic yield shortly after lead fixation due to the decrease of myocardial COI after the implantation over time. If the mentioned excitability difference is not significant between the Conduction System and the myocardium, that results in the lack of transition during the threshold test, even in the presence of effective Conduction System capture. In such cases, additional methods can be used to demonstrate direct capture of the LBB or its fascicles [11, 13, 20, 23, 24, 25].

Measurement of V6RWPT is performed by the pacing stimulus to the peak of the R wave in V6, representing the activation of the left ventricle. As V6RWPT values of LBBP and LVSP are overlapping, any cut‐off values have a trade‐off between sensitivity and specificity. On top of that, V6RWPT has been mainly studied in patients who exhibit a prominent R‐wave in lead V6. Therefore, it remains uncertain whether the same cut‐offs are applicable for patients with an rS pattern in V6 [13].

V6‐V1 interpeak interval reflects activation delay between right and left ventricles and is especially useful for patients with long V6RWPT since it is less influenced by conduction disease compared to V6RWPT, and it can be diagnostic in false‐negative cases, as well. However, it can also be said that it was also studied mainly in patients with prominent R‐wave in lead V6, and it's uncertain whether the same cut‐offs apply to the rS pattern in these leads [13].

Intraoperative visualization of LBB or fascicular potential does not confirm LBB or fascicular pacing by itself [13]. The delay from LBB/fascicular potential to V6RWPT in intrinsic rhythm should correspond to the delay from the pacing stimulus to V6RWPT, allowing for a < 10 ms variability. However, this method is unsuitable for detecting the capture of the Conduction System during follow‐up.

### 4.2 Kinetics of QRS Transition Among Patients With LBB Capture

Our findings demonstrated a significant reduction in the presence of QRS transition over time. Intraoperatively, QRS transition from ns‐ to s‐LBBP or LVSP was observed in 86.4% of patients. However, this detectability decreased markedly in the postoperative period to 47.0% (*p* < 0.001) and further declined to 33.0% (*p* < 0.001) at the first follow‐up. These results highlight a high detectability of QRS transition intraoperatively, and a notable reduction in the detectability of QRS transition, likely influenced by decreasing myocardial COI over time.

Su et al., in a large single‐center study investigating the long‐term safety use of LBBP, found that despite high intraoperative detectability (75.4%), QRS transition recognition is significantly reduced at 3‐month follow‐up (30.9%) in patients receiving s‐LBBP [16]. However, a recent article by Shimeno et al. reported a high 1‐year detectability rate of QRS transition (88.0%) among LBBAP patients who exhibited QRS transition intraoperatively [17]. In contrast, our research findings indicate a lower detectability rate of QRS transition at the 3‐month follow‐up.

Several factors may contribute to this discrepancy, including differences in patient selection (e.g., differences in sex, age, and comorbidities such as ischemic heart disease), procedural indications, implantation techniques, and time elapsed since implantation. In addition, tissue remodeling after lead implantation may affect the long‐term detectability of the QRS transition by altering the detection characteristics over time.

The variability in detectability rates between studies also highlights the need for standardized follow‐up protocols to assess LBB capture. Differences in methodology and interpretation of threshold testing between centers may have a significant impact on reported results. Larger, multicentre studies are warranted to assess the factors influencing threshold test detectability and to investigate why such variation exists between patient populations in different regions of the world.

### 4.3 Classification of Microdislodgement for LBBAP Patients

Microdislodgement is a known complication among patients with LBBAP, which may arise both during the procedure and follow‐up. According to the 2023 Consensus Statement, it is considered an underreported event during follow‐up.

Our classification of microdislodgement into three types (loss of LBB capture, loss of LBBAP with DSP, and RVSP) provides a structured approach to understanding the impact of microdislodgement on pacing outcomes.

### 4.4 Causes of the Disappearance of QRS Transition in Patients Receiving LBBP

We were able to perform threshold tests in 100 of the 118 LBBP cases (84.7%) at all three time points. Our findings showed that despite the loss of QRS transitions among patients with LBBP (52 of 100 patients; 52.0%), the capture of LBB remained in 92 of 100 patients (92.0%). Microdislodgement occurred only in 5.0% of cases, while in the remaining cases (3.0%), macrodislodgement was observed. Although the rates of micro‐ and macrodislodgement may seem high, it is important to note that these data concerns only the subset of 100 LBBP patients with complete threshold test data, not the entire LBBAP population. When considering all the 155 LBBAP patients, microdislodgement occurred in 5 cases (3.2%) and macrodislodgement in 3 cases (1.9%). Microdislodgement should be evaluated by other methods, such as QRS morphology and duration, measurement of V6RWPT or V6‐V1 interpeak interval because the detectability of QRS transition has a significantly lower diagnostic yield postoperatively and during follow‐ups than intraoperatively.

## 5 Limitations

This study involved LBBAP patients from a single‐center cohort; therefore, further external validation is required for our findings. Among the 118 LBBP patients, complete threshold test data at all three time points were available for 100 individuals. In the remaining 18 LBBP cases, data were missing for at least one of the time points due to technical or procedural limitations. However, no statistically significant differences were observed during the sensitivity analysis regarding age, sex, LVEF, QRS width, comorbidities, or implantation duration, suggesting that excluding these cases is unlikely to have introduced systematic bias. Due to the absence of clear guideline recommendations, patient inclusion for LBBAP was based on individual consideration by the implanting physicians.

## 6 Conclusion

This study evaluates QRS transition kinetics in LBBP patients. Intraoperatively, QRS transition shows a high detectability, which decreases significantly during follow‐up. The decline is mainly due to decreasing myocardial COI over time rather than microdislodgement, which occurred only in a minority of cases with loss of transition over time. Our classification of microdislodgement into three types (loss of LBB capture, loss of LBBAP with DSP, and RVSP) aids in understanding its impact on pacing outcomes. Given the reduced diagnostic yield of QRS transition postoperatively, additional methods are essential for diagnosing microdislodgement. This comprehensive approach ensures better long‐term pacing outcomes by addressing the limitations of each method and providing a robust framework for clinical practice and follow‐up in LBBAP patients.

## Author Contributions

All authors attest they meet the current ICMJE criteria for authorship.

## Conflicts of Interest

G.D. reports research grants to institutions from Medtronic and Biotronik and lecture and advisory fees from Medtronic, Biotronik, and Abbott. B.P. reports consulting, lecture fees, nonfinancial support from Abbott, Biosense Webster, Biotronik, Medtronic, Novartis, and Replantmed. P.B. reports lecture fees from Biotronik. The other authors declare no conflicts of interest for this contribution.

## Supporting information

Supplementary document.

## Data Availability

The data that support the findings of this study are available on request from the corresponding author. The data are not publicly available due to privacy or ethical restrictions.

## References

[1] W. Huang , L. Su , S. Wu , et al., “A Novel Pacing Strategy With Low and Stable Output: Pacing the Left Bundle Branch Immediately Beyond the Conduction Block,” Canadian Journal of Cardiology 33, no. 12 (2017): 1736 e1–1736.e3. 10.1016/j.cjca.2017.09.013 29173611

[2] W. Huang , X. Chen , L. Su , S. Wu , X. Xia , and P. Vijayaraman , “A Beginner's Guide to Permanent Left Bundle Branch Pacing,” Heart Rhythm: The Official Journal of the Heart Rhythm Society 16, no. 12 (2019): 1791–1796. 10.1016/j.hrthm.2019.06.016 31233818

[3] D. L. Lustgarten , P. S. Sharma , and P. Vijayaraman , “Troubleshooting and Programming Considerations for His Bundle Pacing,” Heart Rhythm: The Official Journal of the Heart Rhythm Society 16, no. 5 (2019): 654–662. 10.1016/j.hrthm.2019.02.031 31036247

[4] S. S. Ponnusamy , V. Arora , N. Namboodiri , V. Kumar , A. Kapoor , and P. Vijayaraman , “Left Bundle Branch Pacing: A Comprehensive Review,” Journal of Cardiovascular Electrophysiology 31, no. 9 (2020): 2462–2473. 32681681 10.1111/jce.14681

[5] E. Özpak , F. Van Heuverswyn , F. Timmermans , and J. De Pooter , “Feasibility and Safety of Left Bundle Branch Area Pacing in Patients With Septal Hypertrophy,” Journal of Cardiovascular Electrophysiology 34, no. 11 (2023): 2255–2261. 37717221 10.1111/jce.16073

[6] J. Gao , B. Zhang , N. Zhang , M. Sun , and R. Wang , “The Electrocardiogram Characteristics and Pacing Parameters of Permanent Left Bundle Branch Pacing: A Systematic Review and Meta‐Analysis,” Journal of Interventional Cardiac Electrophysiology 63, no. 1 (2022): 215–224. 34173915 10.1007/s10840-021-01000-3

[7] P. S. Sharma , N. R. Patel , V. Ravi , et al., “Clinical Outcomes of Left Bundle Branch Area Pacing Compared to Right Ventricular Pacing: Results From the Geisinger‐Rush Conduction System Pacing Registry,” Heart rhythm: The Official Journal of the Heart Rhythm Society 19, no. 1 (2022): 3–11. 10.1016/j.hrthm.2021.08.033 34481985

[8] S. K. Padala , V. M. Master , M. Terricabras , et al., “Initial Experience, Safety, and Feasibility of Left Bundle Branch Area Pacing,” JACC: Clinical Electrophysiology 6, no. 14 (2020): 1773–1782. 33357573 10.1016/j.jacep.2020.07.004

[9] S. K. Padala and K. A. Ellenbogen , “Left Bundle Branch Pacing Is the Best Approach to Physiological Pacing,” Heart Rhythm O2 1, no. 1 (2020): 59–67. 34113859 10.1016/j.hroo.2020.03.002 PMC8183895

[10] C. W. Israel , S. Tribunyan , and M. Kalyani , “His Bundle Pacing: Troubleshooting at Implantation,” Herzschrittmachertherapie + Elektrophysiologie 31, no. 2 (2020): 160–176. 32399642 10.1007/s00399-020-00690-y

[11] M. Jastrzębski , P. Moskal , A. Bednarek , et al., “Programmed Deep Septal Stimulation: A Novel Maneuver for the Diagnosis of Left Bundle Branch Capture During Permanent Pacing,” Journal of Cardiovascular Electrophysiology 31, no. 2 (2020): 485–493. 31930753 10.1111/jce.14352

[12] M. Jastrzębski , G. Kiełbasa , O. Cano , et al., “Left Bundle Branch Area Pacing Outcomes: The Multicentre European MELOS Study,” European Heart Journal 43, no. 40 (2022): 4161–4173. 35979843 10.1093/eurheartj/ehac445 PMC9584750

[13] H. Burri , M. Jastrzebski , Ó. Cano , et al., “EHRA Clinical Consensus Statement on Conduction System Pacing Implantation: Endorsed by the Asia Pacific Heart Rhythm Society (APHRS), Canadian Heart Rhythm Society (CHRS), and Latin American Heart Rhythm Society (LAHRS),” Europace: European Pacing, Arrhythmias, and Cardiac Electrophysiology: Journal of the Working Groups on Cardiac Pacing, Arrhythmias, and Cardiac Cellular Electrophysiology of the European Society of Cardiology 25, no. 4 (2023): 1208–1236. 37061848 10.1093/europace/euad043 PMC10105878

[14] Á. Estévez Paniagua , S. Briongos Figuero , A. Sánchez Hernández , and R. Muñoz Aguilera , “Left Bundle Fascicular Versus Left Bundle Trunk Pacing: A Comparison of Their Electrical Synchrony Parameters,” Indian Pacing and Electrophysiology Journal 24, no. 5 (2024): 239–246, 10.1016/j.ipej.2024.07.006. 39084520 PMC11480846

[15] J. P. Shroff , A. Nair , L. Q. Tuan , et al., “Electrocardiographic Predictors of Clinical Outcomes in Nonischemic Cardiomyopathy Patients With Left Bundle Branch Area Pacing Cardiac Resynchronization Therapy,” Heart Rhythm: The Official Journal of the Heart Rhythm Society 22 (2024): 1523–1532. 10.1016/j.hrthm.2024.09.018 39278609

[16] L. Su , S. Wang , S. Wu , et al., “Long‐Term Safety and Feasibility of Left Bundle Branch Pacing in a Large Single‐Center Study,” Circulation: Arrhythmia and Electrophysiology 14, no. 2 (2021): e009261. 33426907 10.1161/CIRCEP.120.009261

[17] K. Shimeno , N. Matsumoto , S. Tamura , et al., “Durability of Output‐Dependent QRS Transition and Left Bundle Branch Capture in Left Bundle Branch Area Pacing,” Heart Rhythm: The Official Journal of the Heart Rhythm Society 22 (2024): 1289–1297. 10.1016/j.hrthm.2024.08.039 39181484

[18] X. Liu , H. Niu , M. Gu , et al., “Contrast‐Enhanced Image‐Guided Lead Deployment for Left Bundle Branch Pacing,” Heart Rhythm: The Official Journal of the Heart Rhythm Society 18, no. 8 (2021): 1318–1325. 10.1016/j.hrthm.2021.04.015 33887449

[19] M. Jastrzębski , G. Kiełbasa , P. Moskal , et al., “Fixation Beats: A Novel Marker for Reaching the Left Bundle Branch Area During Deep Septal Lead Implantation,” Heart Rhythm: The Official Journal of the Heart Rhythm Society 18, no. 4 (2021): 562–569. 10.1016/j.hrthm.2020.12.019 33359876

[20] M. Jastrzębski , “ECG and Pacing Criteria for Differentiating Conduction System Pacing From Myocardial Pacing,” Arrhythmia & Electrophysiology Review 10, no. 3 (2021): 172–180. 34777822 10.15420/aer.2021.26 PMC8576513

[21] G. Karwiky , W. Kamarullah , R. Pranata , C. Achmad , and M. Iqbal , “A Meta‐Analysis of the Distance Between Lead‐Implanted Site and Tricuspid Valve Annulus With Postoperative Tricuspid Regurgitation Deterioration in Patients With Left Bundle Branch Area Pacing,” Journal of Cardiovascular Electrophysiology 35, no. 11 (2024): 2220–2229. 39327904 10.1111/jce.16444

[22] S. Wu , X. Chen , S. Wang , et al., “Evaluation of the Criteria to Distinguish Left Bundle Branch Pacing From Left Ventricular Septal Pacing,” JACC: Clinical Electrophysiology 7, no. 9 (2021): 1166–1177. 33933414 10.1016/j.jacep.2021.02.018

[23] M. Jastrzębski , G. Kiełbasa , K. Curila , et al., “Physiology‐Based Electrocardiographic Criteria for Left Bundle Branch Capture,” Heart Rhythm: The Official Journal of the Heart Rhythm Society 18, no. 6 (2021): 935–943. 10.1016/j.hrthm.2021.02.021 33677102

[24] M. Jastrzębski , H. Burri , G. Kiełbasa , et al., “The V6‐V1 Interpeak Interval: A Novel Criterion for the Diagnosis of Left Bundle Branch Capture,” EP Europace 24, no. 1 (2022): 40–47. 10.1093/europace/euab164 PMC8742628 34255038

[25] S. S. Ponnusamy and P. Vijayaraman , “Evaluation of Criteria for Left Bundle Branch Capture,” Cardiac Electrophysiology Clinics 14, no. 2 (2022): 191–202. 35715077 10.1016/j.ccep.2021.12.011

[26] Ó. Cano , P. Jover , H. D. Ayala , et al., “Left Bundle Branch Pacing Versus Left Ventricular Septal Pacing as a Primary Procedural Endpoint During Left Bundle Branch Area Pacing: Evaluation of Two Different Implant Strategies,” Journal of Cardiovascular Electrophysiology 35, no. 1 (2024): 120–129. 37962088 10.1111/jce.16128

[27] J. Lin , Q. Hu , K. Chen , et al., “Relationship of Paced Left Bundle Branch Pacing Morphology With Anatomic Location and Physiological Outcomes,” Heart Rhythm: The Official Journal of the Heart Rhythm Society 18, no. 6 (2021): 946–953. 10.1016/j.hrthm.2021.03.034 33781981

[28] S. Briongos‐Figuero , Á. Estévez‐Paniagua , A. Sánchez‐Hernández , and R. Muñoz‐Aguilera , “Combination of Current and New Electrocardiographic‐Based Criteria: A Novel Score for the Discrimination of Left Bundle Branch Capture,” EP Europace 25, no. 3 (2023): 1051–1059. 36691717 10.1093/europace/euac276 PMC10062292

[29] J. C. Diaz , U. B. Tedrow , M. Duque , et al., “Left Bundle Branch Pacing vs Left Ventricular Septal Pacing vs Biventricular Pacing for Cardiac Resynchronization Therapy,” JACC: Clinical Electrophysiology 10, no. 2 (2024): 295–305. 38127008 10.1016/j.jacep.2023.10.016

[30] K. Curila , L. Poviser , P. Stros , et al., “LVSP and LBBP Result in Similar or Improved LV Synchrony and Hemodynamics Compared to BVP,” JACC: Clinical Electrophysiology 10, no. 7 Pt 2 (2024): 1722–1732. 38829298 10.1016/j.jacep.2024.04.022

[31] H. Zhu , C. Qin , A. Du , et al., “Comparisons of Long‐Term Clinical Outcomes With Left Bundle Branch Pacing, Left Ventricular Septal Pacing, and Biventricular Pacing for Cardiac Resynchronization Therapy,” Heart Rhythm: The Official Journal of the Heart Rhythm Society 21, no. 8 (2024): 1342–1353. 10.1016/j.hrthm.2024.03.007 38461922

