# Supplementary material for: Kinetics and Disappearance of QRS Transition in Patients Undergoing Left Bundle Branch Pacing – A Novel Method for Classifying Microdislodgement
Source: J Cardiovasc Electrophysiol. 2025 Jul 11;36(9):2254–63. doi: 10.1111/jce.16779 (PMC12420859; doi:10.1111/jce.16779)
Supplement: Supplementary file 1 — Supplementary document. [file JCE-36-2254-s001.docx]

**Supplementary Figure S1**

Diagnostic steps for confirmation of LBB capture during the implantation (N=118)

Values are given as n (%). This figure illustrates the diagnostic pathway for confirming left bundle branch (LBB) capture during implantation. In our study, LBB capture was considered verified when the pacing lead successfully reached the left bundle branch area (LBBA) and at least one of the predefined diagnostic criteria was fulfilled. LBBB = left bundle branch block; NIVCD = nonspecific interventricular conduction disease; RBBB = right bundle branch block; V6RWPT = V6 R wave peak time.

# Supplementary Table S1. Sensitivity analysis for baseline and procedural characteristics of LBBP patients with Complete (n=100) vs. Incomplete (n=18) threshold test data

| **Clinical variable** | Complete threshold test data (n=100) | Incomplete threshold test data (n=18) | p-value |
| --- | --- | --- | --- |
| Male | 62 (62.0) | 15 (83.3) | 0.108 |
| Hypertension (HT) | 84 (84.0) | 16 (88.9) | 0.736 |
| Diabetes (DM) | 39 (39.0) | 7 (38.9) | 1.000 |
| Ischaemic cardiomyopathy (ICM) | 35 (35.0) | 8 (44.4) | 0.440 |
| Chronic kidney disease (CKD) | 31 (31.0) | 8 (44.4) | 0.285 |
| LVEF (%) | 47.7 ± 14,4 | 42.5 ± 13.6 | 0.130 |
| Preoperative QRS width(ms) | 118.0 ± 30.2 | 131.0 ± 40.1 | 0.210 |
| Implantation duration (min) | 60.5 ± 31.6 | 64.6 ± 33.3 | 0.507 |

Values are given as mean ± SD or n (%). This sensitivity analysis comparing baseline and procedural characteristics between patients with complete (threshold test data at all three-time points; n=100) and incomplete data (missing data at any time point; n=18) revealed no statistically significant differences, suggesting that the exclusion of these cases is unlikely to have introduced systematic bias
